# DispHred: A Server to Predict pH-Dependent Order–Disorder Transitions in Intrinsically Disordered Proteins

**DOI:** 10.3390/ijms21165814

**Published:** 2020-08-13

**Authors:** Jaime Santos, Valentín Iglesias, Carlos Pintado, Juan Santos-Suárez, Salvador Ventura

**Affiliations:** Institut de Biotecnologia i Biomedicina and Departament de Bioquímica i Biologia Molecular, Universitat Autònoma de Barcelona, 08193 Barcelona, Spain; jaime.santos@uab.es (J.S.); valentin.iglesias@uab.cat (V.I.); Carlos.pintado@e-campus.uab.cat (C.P.); juansans98@gmail.com (J.S.-S.)

**Keywords:** intrinsically disordered proteins, pH, bioinformatics, disorder prediction, conditional folding, machine learning

## Abstract

The natively unfolded nature of intrinsically disordered proteins (IDPs) relies on several physicochemical principles, of which the balance between a low sequence hydrophobicity and a high net charge appears to be critical. Under this premise, it is well-known that disordered proteins populate a defined region of the charge–hydropathy (C–H) space and that a linear boundary condition is sufficient to distinguish between folded and disordered proteins, an approach widely applied for the prediction of protein disorder. Nevertheless, it is evident that the C–H relation of a protein is not unalterable but can be modulated by factors extrinsic to its sequence. Here, we applied a C–H-based analysis to develop a computational approach that evaluates sequence disorder as a function of pH, assuming that both protein net charge and hydrophobicity are dependent on pH solution. On that basis, we developed DispHred, the first pH-dependent predictor of protein disorder. Despite its simplicity, DispHred displays very high accuracy in identifying pH-induced order/disorder protein transitions. DispHred might be useful for diverse applications, from the analysis of conditionally disordered segments to the synthetic design of disorder tags for biotechnological applications. Importantly, since many disorder predictors use hydrophobicity as an input, the here developed framework can be implemented in other state-of-the-art algorithms.

## 1. Introduction

Intrinsically disordered proteins (IDPs) are a class of polypeptides that do not require a defined folded structure to execute their biological activities [1,2,3]. The plasticity of these biomolecules allows them to interact with structurally diverse partners, and they are often involved in the wiring of protein networks, acting both as central hubs and as molecular switches [4]. The unfolded nature of IDPs is intrinsically encoded in their primary sequence, which is generally enriched in ionizable and polar residues and depleted of hydrophobic amino acids [5]. Thus, IDPs’ extended conformation depends both on electrostatic repulsions between uncompensated charges and on a low hydrophobicity, which prevents extensive protein compaction [6].

Based on the balance between attractive and repulsive forces in IDPs, Uversky and coworkers proposed that they populated a distinct region in the mean net charge–hydropathy (C–H) phase space diagram and demonstrated that an empirical boundary line was enough to discriminate folded and disordered proteins [6]. Under that premise, the disordered nature of a polypeptide sequence can be predicted by evaluating its C–H relationship in the aforementioned attraction–repulsion scheme. The C–H plot analysis has been applied for disorder prediction; it lies behind the popular FoldIndex algorithm [7], and it is also computed by other multiparametric software [8].

More than 50 predictors, based on different molecular principles, have been developed to assess protein disorder, thus providing a robust toolbox for identifying natively unfolded proteins or their regions [8,9,10]. Besides, new tools able to reverse-engineer the above-mentioned principles into a sequence that now allows for the artificial design of disordered protein segments [11,12]. Nevertheless, most of these methods are blind to the protein context, even if IDPs are extremely sensitive to environmental fluctuations [13,14]. Ligands, binding partners, or solvent conditions such as ions concentration or pH, have been reported to induce conditional folding in IDPs [6,15,16]. Therefore, it is surprising to find out that those effects have been mostly disregarded in state-of-the-art computational approaches. Indeed, it is immediately evident that the C–H relationship of a given protein is not constant since both protein net charge and hydrophobicity can be modulated by factors that are extrinsic to the sequence.

In recent work, we showed that the solution’s pH effect on IDPs solubility is not restricted to its effect on the charge of ionizable residues since the pH also modulates the sequence hydrophobicity [17], a traditionally neglected effect. Driven by this simple idea, we revisit here the C–H concept, on the evidence that both protein net charge and hydrophobicity are dependent on pH. By delineating a boundary condition similar to the one described by Uversky [6], we demonstrate that IDPs’ pH-induced folding can be predicted just by evaluating the pH dependence of the C–H space diagram. This allowed us to develop DispHred, a first computational approach to predict protein disorder as a function of pH. DispHred is freely available for academic users at https://ppmclab.pythonanywhere.com/DispHred. We hope that the data we present here may prompt the development of a new generation of disorder predictors that include solvent conditions on their pipelines.

## 2. Results

### 2.1. Validation of a pH-Dependent Hydropathy Scale for C–H Plot-Based Predictions

The original C–H analysis was developed using the Kyte–Doolittle hydropathy scale to calculate the mean hydrophobicity of protein sequences [6,7,18]. Here, we implement a novel amino acid pH-dependent hydropathy scale developed by Zamora and co-workers [19], based on implicit solvation calculations, that allow us to evaluate the effect of the solution pH on sequence hydrophobicity. As a first step in developing our approach, we needed to assess the performance of this pH-dependent scale for C–H plot-based order–disorder predictions at neutral pH. Uversky and Dunker did an extensive analysis of 19 diverse hydropathy scales to compare their performance in C–H plot-based predictions [20]. They reported that the Guy hydropathy scale [21] had the highest discriminative power, while Kyte–Doolittle performance was in the average of the 19 scales. Additionally, they developed a new scale that provided the best order–disorder discrimination (IDP–Hydropathy) [20].

**Figure 1:**
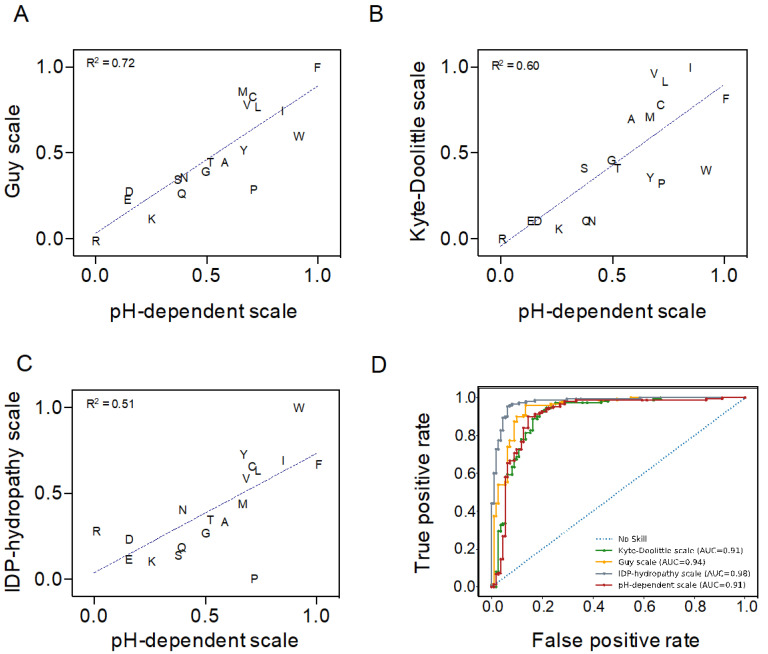
Comparison of four different hydropathy scales at pH 7.0. Correlation between pH-dependent scale and (**A**) Guy, (**B**) Kyte–Doolittle, and (**C**) IDP–hydropathy scales. Letters correspond to the amino acid one-letter code. Hydropathy values are normalized between 0 and 1, corresponding to the minimum and maximum values for each scale. The R^2^ value of the linear regression is shown in each graph. (**D**) Receiver Operating Characteristic curves showing the performance of the four scales in discriminating a dataset of fully disordered (*n* = 111) and single-chain folded (*n* = 150) proteins.

We compared the pH-dependent hydropathy (pH-dependent) scale with the Kyte–Doolittle, Guy, and IDP–Hydropathy scales. First, we normalized the four scales between 0 and 1, assigning a value of 1 to the highest hydrophobicity. Then, we calculated the values for the pH-dependent scale at pH 7.0. We found the highest correlation with the Guy scale (R^2^ = 0.72), followed by the Kyte–Doolittle (R^2^ = 0.60) and the IDP–hydropathy (R^2^ = 0.51) scales (Figure 1A–C). The correlation between the Guy and Kyte–Doolittle scales is R^2^ = 0.78. In contrast, as it happens for the pH-dependent scale, the correlation between the IDP–hydropathy and the Guy or the Kyte–Doolittle scales is low, with R^2^ = 0.52 and R^2^ = 0.33, respectively. These low correlations stem mostly from the fact that, counter-intuitively, the IDP–hydropathy scale considers Pro as the most hydrophilic residue, with a value of 0 in our normalized scale. Removing Pro from the correlation between the pH-dependent and IDP–hydropathy scales increases R^2^ to 0.70 and arbitrarily assigning this residue a value of 0 in the pH-dependent scale (pH–Pro-corrected scale) results in an R^2^ = 0.74 (Supplementary Figure S1A).

We next ensembled a dataset of 111 experimentally validated fully disordered proteins and 150 folded single-chain proteins with X-ray resolved structures (Supplementary Table S1) to test the discriminatory power of the four scales in a C–H plot analysis. The ability to classify ordered and disordered sequences of each scale was assessed by applying a Receiver Operating Characteristic (ROC) method. The associated area under the curve (AUC) was used as a sensitivity–specificity reporter. The pH-dependent and the Kyte–Doolittle scales showed an identical discriminatory potential (AUC = 0.91), while the Guy and IDP–hydropathy scales demonstrated slightly higher performances (AUC = 0.94 and 0.98, respectively) (Figure 1D). The pH–Pro-corrected scale exhibited an AUC = 0.95 (Supplementary Figure S1B), which suggests that the minimal value assigned to Pro in the IDP–hydropathy scale contributes to its higher discrimination.

Overall, the analysis suggested that the pH-dependent scale compared well with the other analyzed scales at pH 7.0, with a discriminatory power identical to the widely employed Kyte–Doolittle scale. Thus, this scale will allow us to extend the C–H predictive potential to the full pH scale without compromising the performance at neutral pH significantly. Despite its higher discrimination, we preferred to not use the pH–Pro-corrected scale and keep the hydropathy value obtained from implicit solvation calculations for Pro residues [19].

### 2.2. C–H Space Phase Diagram and Order–Disorder Boundary Condition Can Anticipate pH-Induced Order–Disorder Transition of IDPs

**Figure 2:**
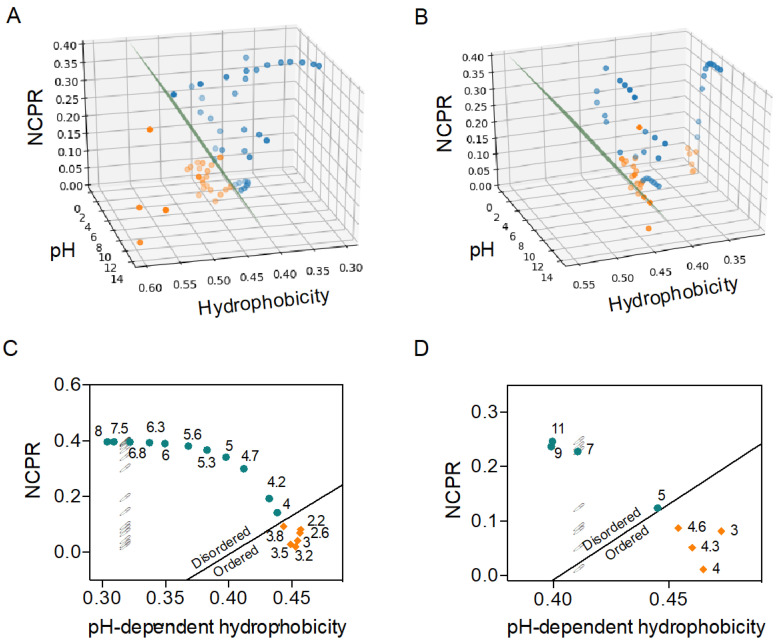
Charge–Hydropathy-based analysis of pH modulated order–disorder transitions. Three-dimensional C–H plots containing 59 datapoints of 7 proteins at different pHs, computing pH influence over (**A**) sequence net charge per residue (NCPR) and hydrophobicity, or (**B**) assuming constant hydrophobicity values (as calculated at pH 7.0). Blue and orange points correspond to conditions in which protein/peptides are disordered and folded, respectively. The green surfaces delimit the boundary conditions between folded and disordered proteins defined by Equation (1). (**C**,**D**) Two-dimensional C–H plots of (**C**) prothymosin and (**D**) PEST-c-myc using the same color pattern than in panels A and B for folded-unfolded datapoints. A solid line represents the boundary condition. Open circles represent the same data points assuming constant hydrophobicity values (as calculated at pH 7.0).

Next, we explored whether the C–H model would be a reliable tool to predict the pH-dependent order–disorder transition in IDPs. To that end, we performed a bibliographic search of structural data on IDPs that suffer a conditional folding at specific pHs. We collected 59 bibliographic pH datapoints for 7 disordered proteins and peptides (Figure 2 and Supplementary Figure S2). For each point, we calculated the protein net charge per residue (NCPR) and protein mean hydrophobicity at the given pH <H_pH_>. To do so, NCPR is calculated using the Henderson–Hasselbach equation, and <H_pH_> is computed according to the pH-dependent scale developed by Zamora and co-workers [19]. We plotted each datapoint in a 3-axis scatter plot according to its pH, <H_pH_> and NCPR, with the dot’s color indicating whether the protein was folded or disordered in this condition (Figure 2).

To develop a consistent C–H-based order–disorder classification for the experimental data, we sought to seek the order–disorder boundary condition that allowed the maximal separation between the two states. Since the datasets for the different proteins diverged in size, nature, and source, we assumed that a classic iterative analysis might lead to overfitting and/or result in a biased boundary condition in case some data points were misclassified.

To minimize such limitations, we applied a support vector machine (SVM) learning strategy, a supervised feedforward network specifically designed to build a binary classifier and retrieve the boundary condition that maximizes the separation between observations [22,23]. SVM-based analysis reduces overfitting and tolerates a certain degree of misclassified data points without forcing a bias, being robust classification strategies, and increasing their predictive potential when applied to new observations, especially near the boundary condition. Additionally, since SVM analysis takes into account a slight uncertainty and misclassification, it also provides a margin near the boundary line (dashed lines in Supplementary Figure S3) that can be used as a confidence interval in a subsequent classification of new data points in predictive applications.

By using the above-described SVM-based analysis, we identified a linear boundary condition defined by Equation (1)

(1) $$Dis_{pH}=2.775<H_{pH}>-\left|NCPR\right|-1.118$$

that successfully discriminates between folded and disordered proteins with a Matthews Correlation Coefficient of 0.97 (Supplementary Figure S3A, Table 1). Note that our boundary condition is reasonably similar to that previously defined by Uversky and co-workers (Equation (2)) [6] for order–disorder classifications at neutral pH.

(2) I=2.785<H>−|<R>|−1.151

<*H*> and <*R*> correspond to the mean hydrophobicity and mean charge at neutral pH, respectively.

In contrast, applying the same SVM analysis but considering that hydrophobicity is independent of pH, we did not observe a consistent classification of the datapoints—Matthews Correlation Coefficient of 0.6—and the boundary line didn’t satisfy the C–H relationship (Supplementary Figure S3B, Supplementary Table S2).

As shown in Figure 2A, the boundary plane defined by Equation (1) satisfactorily delimitated folding–unfolding transitions for the analyzed IDPs, with only one datapoint wrongly predicted and still reasonably close to the boundary. This translates into 98% accuracy in predicting the proteins’ conformational states at any given pH (Table 1). On the contrary, by considering that hydrophobicity is independent of pH (and computing its value at pH 7.0 and under the same boundary condition as Equation (1)), we observed that the NCPR change alone could not discriminate between folded and disordered sequences (Figure 2B and Table 1). This observation evidences the importance of modeling the pH-dependent hydrophobicity when predicting protein disorder.

Prothymosin is a classic example of an IDP at neutral pH that experiences a conditional folding at low pHs, characterized by the gain of α-helical structure [24]. The transition occurs between pH 3.5 and pH 5.0, with prothymosin being fully folded below pH 3.5 and fully unfolded above pH 5.0. In a two-dimensional projection of the data points for this protein, we can observe that all folded points fall below the boundary line, being thus accurately predicted (Figure 2C). We also observed that our pH-dependent C–H representation also succeeds in delineating the transition range (pH 3.5–5). Similarly, the disordered PEST region (201—268) from human c-Myc oncoprotein collapses into a folded conformation at pHs below 4.8 [25], a transition that is successfully identified by our pH-dependent C–H ratio (Figure 2D). Note that the same analysis considering a constant hydrophobicity is blind to these structural conversions (open circles in Figure 2C,D). The same trend can be observed in the two-dimensional C–H plots of the other five protein sets in Figure 2A,B (Supplementary Figure S2).

These data demonstrate that the effect of pH on IDPs’ conditional folding can be successfully predicted by applying a pH-dependent C–H analysis. With these results in hand, we aimed to develop a computational tool for predicting protein disorder that considers implicitly the solution pH, which we named DispHred.

### 2.3. Rational and Implementation of DispHed, a pH-dependent Predictor of Sequence Disorder

DispHred uses the C–H space diagram analysis proposed by Uversky and co-workers and later implemented in FoldIndex [6,7]. Nevertheless, instead of considering constant net charges and hydrophobicity for each analyzed sequence, DispHred assumes that the solution pH modulates both parameters. Thus, DispHred computes the protein NCPR and the mean hydrophobicity of a sequence as a function of pH. Then, DispHred applies the boundary condition defined by Equation (1) to separate folded and disordered proteins. Dis_pH_ positive values correspond to sequences classified as folded and negative values to those classified as disordered at the analyzed pH or pH range. The SVM approach provides a margin of ± 0.02 around the boundary line used as a confidence interval in the classification.

DispHred calculates the Dis_pH_ score for all the analyzed pHs, profiling the pH-dependence disorder of a protein sequence, and thus including the pH dimension in the classical C–H phase diagram. DispHred runs a user-defined sliding window that enables the analysis of the folded/disordered regions in a protein sequence at every requested pH. Sequence stretches fall in three classes: i) regions that are predicted to be always folded in the analyzed pH interval, ii) regions that are predicted to be always disordered in this pH interval, and iii) regions whose folded/disordered conformation is modulated by the pH.

**Figure 3:**
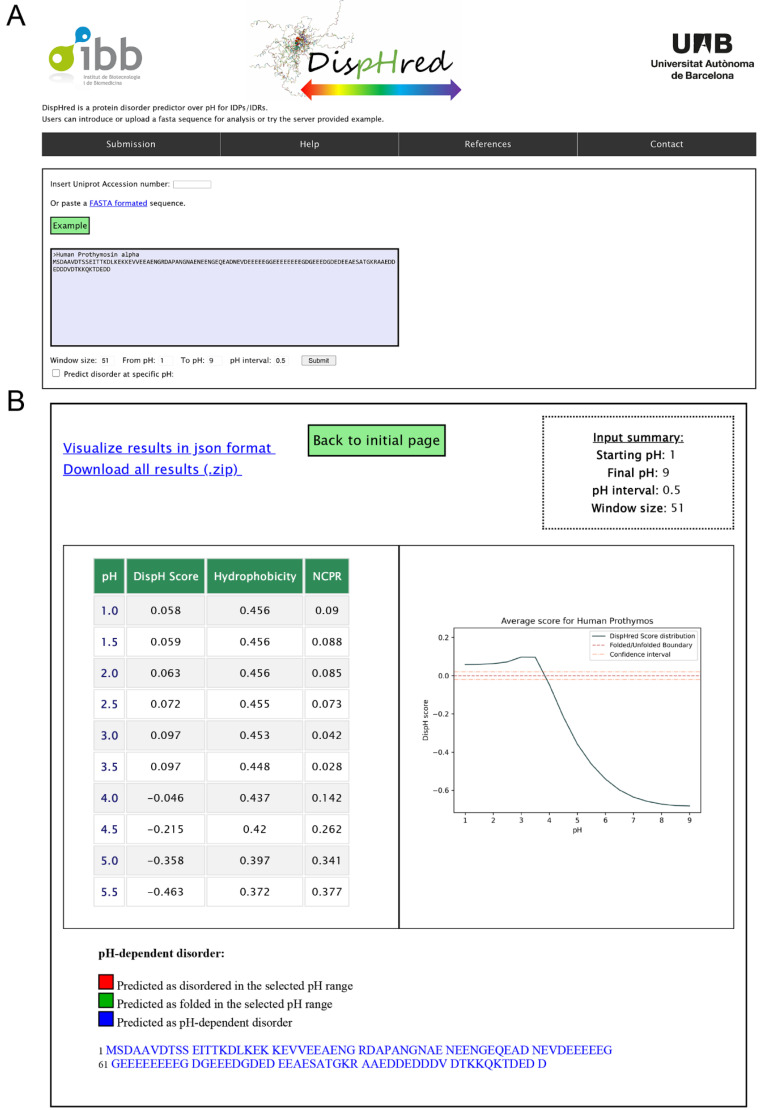
DispHred web server interface. (**A**) Web input page. The user can paste their sequence or insert a valid Uniprot Accession number. DispHred works by default by checking disorder in a range of pHs but allows users to test values at a specific pH. By default, a 51-residue sliding window is populated, but users can personalize its length. (**B**) Web results page for a selected range of pHs. Two clickable links appear on the upper left part of the screen with a JSON file or a ZIP file containing DispHred calculations and generated figures. On the lower left part, the table shows the DispHred, hydrophobicity, and NCPR average scores for each pH. Clicking each pH will open a figure representing the Dis_pH_ score variation along the sequence for the selected pH. On the lower right part, a figure representing the Dis_pH_ average score for each pH is shown. Scores above the red dashed line indicate predicted order. On the bottom of the screen, folded, disordered, and conditionally disordered regions are indicated in the sequence in green, red and blue, respectively.

DispHred is available at https://ppmclab.pythonanywhere.com/DispHred. DispHred is free for academic users and does not require login. In the input page the user can (i) introduce a sequence in FASTA format or insert a valid Uniprot Accession number, (ii) select the pH range and step size for the analysis or type a single specific pH and (iii) select the sliding window size (Figure 3A). After running the program, the user will be redirected to a results page containing the report of the analysis (Figure 3B): Dis_pH_ scores, mean hydrophobicity, and NCPR for each of the analyzed pHs, a graph showing Dis_pH_ score as a function of pH, and clickable links that redirect to the sequence profile prediction at each desired pH. The protein regions exhibiting pH-dependent and pH-independent folded/disordered conformations are colored on top of the input sequence.

Users can retrieve all data in a JavaScript Object Notation (JSON) file or download all the generated data in a compressed ZIP file. A clickable example is provided in the input page to illustrate DispHred outputs.

## 3. Discussion

Structural disorder is a fundamental trait of protein biology that complements the activities of structured proteins and domains by contributing flexibility and plasticity [26,27,28]. In contrast to folded proteins, IDPs exist as ensembles sampling a wide range of dynamic conformations in which the bulk of the primary sequence is highly exposed to the solvent. Accordingly, IDPs’ properties display little dependence on structural elements and can be inferred from the primary sequence, which has allowed the design of computational tools for predicting, designing, and analyzing protein disorder [8,9,11]. At the same time, IDPs are extremely sensitive to environmental conditions; an effect often disregarded in predictive approaches. Among the different parameters that may affect IDPs’ properties, the solution pH has a significant impact, mainly due to the high prevalence of ionizable residues in these polypeptides [14,15,17,29]. In this work, we demonstrated that the effect of pH on the disordered nature of a protein sequence could be easily predicted by evaluating the changes in protein charge and hydrophobicity as a function of this parameter. Even if the effect of pH over net charge is well-recognized, hydrophobicity is usually considered to be a pH-independent property. However, we found that the evaluation of the pH-dependent hydrophobicity is fundamental for the accuracy of the order/disorder prediction in any given condition.

The analysis of the local or global hydrophobicity of protein sequences is a pivotal stage in many in silico pipelines aimed to predict protein disorder and its associated properties. A significant number of disorder predictors, such as FoldIndex or PONDR, rely on the direct or indirect analysis of hydrophobicity, a property that is also used to predict folding upon binding, RNA–, DNA-interactions or post-translational modification sites in IDPs [7,8,9,30,31,32,33,34]. Thus, the identification of hydropathy scales suitable for such analyses attracted significant attention in the past [20]. Our results indicate that by applying a recently developed pH-dependent hydropathy scale, the contribution of this predictive physicochemical property to disorder prediction can be extended to the full pH scale. Thus, the implementation of pH-dependent hydropathy scales, like the one we use here, may increase applicability in currently available algorithms.

pH, ion concentrations, redox state, or post-translational modifications are known regulators of protein function by controlling the switch between the disordered and folded or partially folded states of polypeptides. Thus, although the conditional disorder’s prediction is a challenging task, it is fundamental to elucidate the functionality of IDPs. [13,35]. To advance in this direction, we developed DispHred, an online web server that exploits the C–H space analysis to predict protein disorder as a function of pH. Its main application is the profiling of protein disorder across a continuous pH interval, demonstrating a high accuracy in classifying the pH-modulated order–disorder transitions for sequentially unrelated model proteins and peptides. Additionally, DispHred allows the assessment of the specific protein regions contributing the most to conditional disorder.

In essence, DispHred is the first disorder predictor dedicated to evaluating the effect of the solution pH and constitutes a proof-of-concept for the implementation of this kind of approach in future predictive endeavors. Intrinsically disorder tags are increasingly used to solubilize proteins and to engineer the pharmacological properties of protein and peptide pharmaceuticals [36]. We envision that DispHred can be of significant help in these biotechnological tasks.

## 4. Materials and Methods

### 4.1. Data Collection

The dataset of 111 experimentally verified fully disordered proteins was obtained from the Disprot database (DisProt 2020_06) [37] by selecting proteins with a 100% disorder coverage. The set of 150 fully folded sequences was randomly extracted from the Protein Data Bank (PBD) under the query single-chain structures larger than 100 residues and determined by X-ray crystallography.

Data regarding the effect of pH on protein disorder was extracted from the bibliography. Data regarding the pH-dependent folding of prothymosin was obtained from the characterization of Uversky and coworkers [24]. Order–disorder pH-transition of the PEST region (201—268) from human c-Myc oncoprotein was analyzed in Mohd. Ziauddin Ansari and Rajaram Swaminathan’s study [25]. LL-37 pH-dependent helix formation was reported by Johansson and coworkers [38]. Victor Muñoz and Luis Serrano reported the effect of solution pH on a model peptide Ac-AKAAKAKAAKAKAAKA-NH2 [39]. Data on the pH-modulated collapse of human histones were extracted from Munishkina and coworkers [40]. The analysis of the disordered A-domain of the Toc132 receptor disorder was performed by Lynn GL Richardson, Masoud Jelokhani-Niaraki, and Matthew D Smith [41]. The conformational fluctuations of the 36-loop region of the influenza hemagglutinin were analyzed by Chavela M. Carr and Peter S. Kim [42].

### 4.2. DispHred: Evaluation of Hydrophobicity and Charge as a Function of pH

To analyze the lipophilicity of protein sequences, we employed the pH-dependent lipophilicity scale developed by Zamora and coworkers. They used continuum solvation calculations, which allow us to calculate the hydrophobicity of a given residue at the desired pH [19]. Then, DispHred uses a sliding window with a user-defined length to calculate the average hydrophobicity in the window and assigns it to the residue in the center. In the analysis performed in this article, we used a fixed window of 7 residues. The results were averaged to calculate the mean hydrophobicity of the sequence at the analyzed pH.

Protein NCPR is calculated by applying the Henderson–Hasselbalch equation to derive the partial charge of each ionizable residue at the analyzed pH. Then, global NCPR is calculated as the sum of all partial charges divided by the protein length. To calculate the Dis_pH_ score of a given window, the NCPR is calculated using the residues included in this particular window and its length.

### 4.3. Hydropathy Scales Performance Analysis at Neutral pH

We delineated a C–H plot for each of the analyzed hydropathy scales. Each scale was normalized from 0 to 1 according to the increased hydrophobicity of the protein residues; for the pH-dependent scale, we employed the values calculated at pH 7.0 [19]. The performance of the different scales was evaluated using a ROC analysis, in which the true-positive rate is plotted against the false-positive rate. The ROC analysis was performed against a dataset of 111 fully disordered proteins and 150 single-chain folded proteins. The AUC was taken as an indicator of sensitivity and sensibility.

### 4.4. Support Vector Machine Analysis

SVM was applied to define the optimal boundary line delimitating two classes of samples as folded or disordered. NCPR and pH-dependent hydrophobicity were calculated as previously stated for the 59 data points. Experimental data was labeled as ordered or disordered as described in the literature and employed for the machine learning process. To perform the analysis, we used the freely available machine learning library scikit-learn [43]. The SVM kernel was set to “linear” to map the data on a two-dimensional space.

### 4.5. DispHred: Prediction of Sequence Disorder

DispHred uses a C–H plot analysis to discriminate between folded and disordered sequences at the analyzed pH by applying a defined boundary condition. For each pH, the mean hydrophobicity (<H_pH_>) and the absolute value of the NCPR are calculated. Then, the Dis_pH_ score is obtained by applying the SVM–derived boundary condition (Equation (1)). Positive and negative values are classified as folded or disordered, respectively. DispHred calculates the Dis_pH_ score at all the pHs in the desired range to profile sequence disorder as a function of pH. DispHred also analyzes the Dis_pH_ score of the sliding windows to identify specific stretches whose disorder is affected by pH.

### 4.6. Performance Analysis

The sensitivity, specificity, precision, accuracy and false discovery rate when predicting order–disorder transitions was evaluated as follows: Sensitivity = TP/(TP + FN); Specificity = TN/(TN + FP); Precision = TP/(TP + FP); Accuracy = (TP + TN)/(TP + TN + FP + FN); and False Discovery Rate = FP/(FP + TP). F1 Score and Matthews Correlation Coefficient were calculated as previously described in [44]. TP, TN, FP and FN correspond to true positives, true negatives, false positives and false negatives, respectively

### 4.7. DispHred Web-Server

The DispHred web server interface was built in HTML/CSS/JS. It uses the Django 3.0 framework working with python 3.7. The figures are generated using matplotlib library [45]. The server is platform-independent, free and open for academic users and does not require a previous login.

## Figures and Tables

**Table 1:** Performance of pH-dependent and pH-independent hydrophobicity approaches in predicting pH-conditioned order–disorder transitions in a C–H analysis by applying Equation (1). Unfolded sequences correctly predicted to be unfolded were classified as true positives. The highest values for each measure are indicated in bold.

Measure	pH-Dependent Hydrophobicity	pH-Independent Hydrophobicity
Sensitivity	**1.00**	**1.00**
Specificity	**0.96**	0.21
Precision	**0.97**	0.65
False Discovery rate	0.03	**0.35**
Accuracy	**0.98**	0.68
F1 Score	**0.99**	0.79
Matthews Correlation Coefficient	**0.97**	0.37

## Supplementary Materials

Supplementary materials can be found at https://www.mdpi.com/1422-0067/21/16/5814/s1.

## Abbreviations

AUC	Area under the curve
C–H	Charge–hydropathy
IDP	Intrinsically disordered protein
NCPR	Net charge per residue
ROC	Receiver Operating Characteristic
SVM	Support Vector Machine
<H>	Mean hydrophobicity
