# Supplementary material for: DispHred: A Server to Predict pH-Dependent Order–Disorder Transitions in Intrinsically Disordered Proteins
Source: Int J Mol Sci. 2020 Aug 13;21(16):5814. doi: 10.3390/ijms21165814 (PMC7461198; doi:10.3390/ijms21165814)
Supplement: Supplementary file 1 [file ijms-21-05814-s001.pdf]

Supplementary Figure S1:

Evaluation of the pH-Pro-corrected hydropathy scale. (A) Correlation between pH-Pro-corrected hydropathy scale and IDP-hydropathy scale. (B) ROC curve analysis of the performance of pH-dependent and pH-Pro-corrected hydropathy scales in discriminating a dataset of a fully disordered (n=111) and single-chain folded (n=150) proteins.

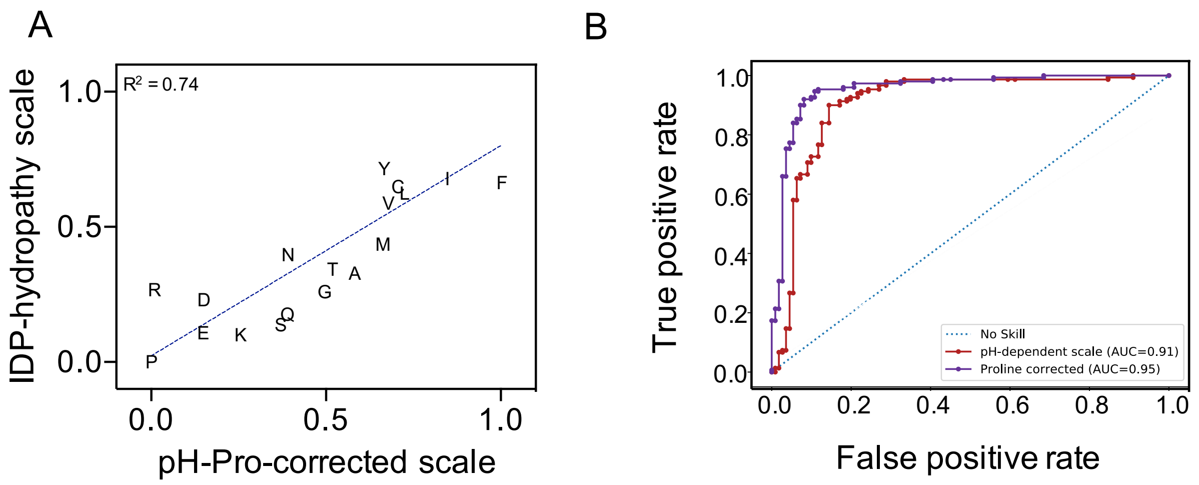

Supplementary Figure S2:

C-H plots of Ac-AKAAKAKAAKAKAAKA-NH2 (**A**), a 36-loop region of the influenza hemagglutinin (**B**), A-domain of the Toc132 receptor (**C**), LL-37 (**D**) and human histones (**E**). Solid line delimits folded-unfolded boundary condition. Blue and orange data points correspond to bibliographically unfolded and folded conditions, respectively. Open circles represent the same points considering a constant hydrophobicity (pH 7).

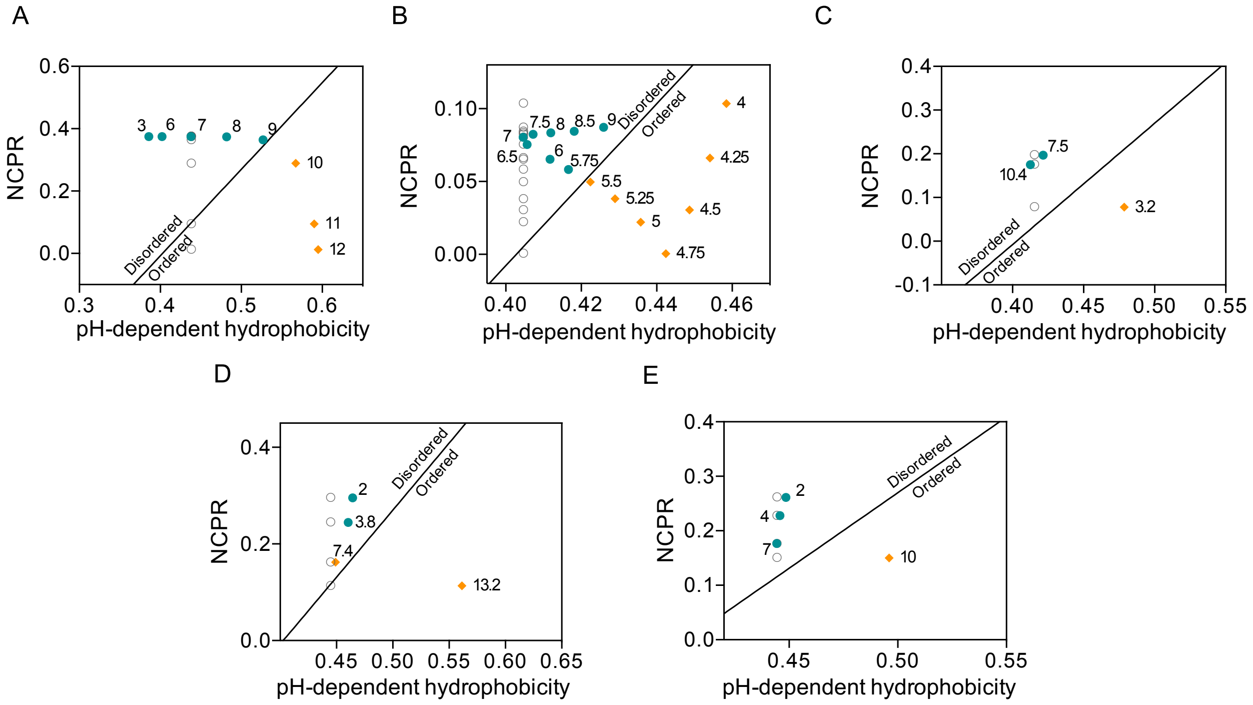

Supplementary Figure S3:

SVM-based classification of pH-conditioned ordered-disordered protein sequences based on their C-H relation. **(A, B)** C-H plots containing 59 datapoints; 35 labeled as disordered (blue) and 24 as folded (orange). Each point is defined by its calculated NCPR and its mean hydrophobicity at their experimental pH (A) or neutral pH (B). The solid line represents the optimal boundary condition, whereas dashed lines delimitate the maximum margin.

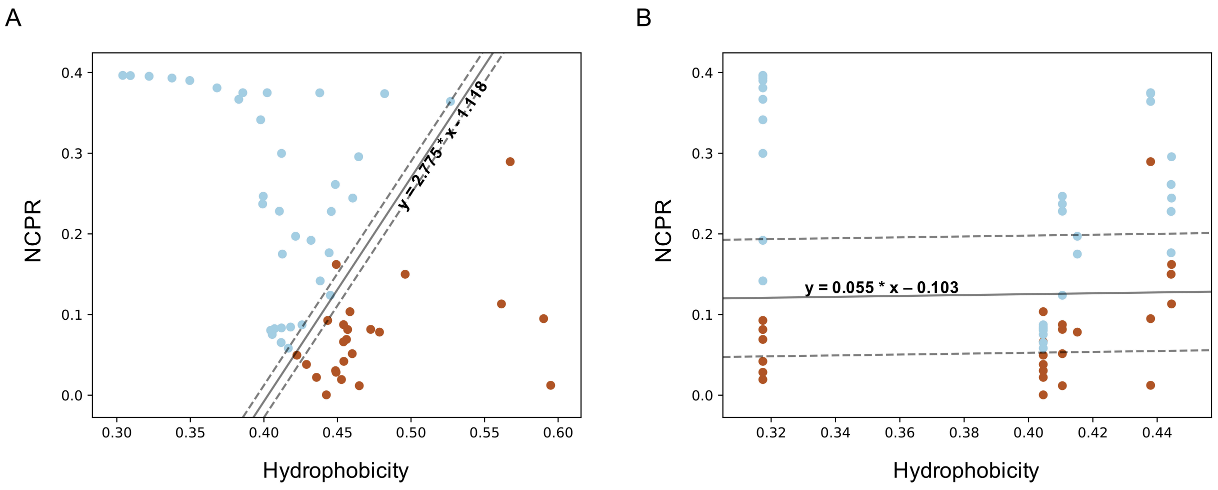

Supplementary Table S1:

111 fully disordered proteins and 150 single-chain folded proteins used to evaluate the discriminatory performance of the hydropathy scales.

Disprot identifier (disorder proteins)	PDB ID (Folded proteins)
DP00667	5RDH
DP00325	2GU9
DP00661	1IDP
DP02205	1NKO
DP01431	5DMD
DP00584	3CJW
DP01946	1RAT
DP00592	1RH9
DP01300	1LZ1
DP01425	1TGN
DP00219	4L1H
DP00068	1WOU
DP00587	3FY3
DP00040	2PAB
DP00871	1R3F
DP00042	5J7E
DP00689	1TGL
DP01945	2FEM
DP00645	2JJF
DP00329	6N8U
DP00878	4JAR
DP00146	1ACX
DP01786	1ALD
DP00663	1BOL
DP01512	21BI
DP00214	1SUR

DP00057	1ILT
DP00041	1J8Y
DP01435	2BLM
DP01146	3CBH
DP00664	2O71
DP01509	1BRY
DP00047	1K2P
DP00877	1ND7
DP00929	2RBL
DP00694	1TIA
DP01521	6EXR
DP00116	1R6F
DP01942	6K3H
DP01087	4YXX
DP02167	1FEW
DP01293	1FIM
DP01088	1FTS
DP00132	1BGT
DP00070	1BSF
DP01677	1PCL
DP01299	1KON
DP01295	1SMT
DP01148	2BTZ
DP00140	3DXF
DP00008	3IF0
DP00657	2GE8
DP00626	5E7I
DP01071	2V71
DP00546	3I4H
DP00534	1QMN
DP01386	3JYM

DP00630	1C79
DP01381	1QDM
DP02066	1WVI
DP00815	1ERK
DP01488	2RIG
DP00253	1U2J
DP01876	2ILA
DP02299	2GZR
DP00112	1H6J
DP01383	1QTR
DP01074	6H2E
DP00005	6RJX
DP00028	2INT
DP01385	4ZWQ
DP00015	5L0O
DP00288	3MFM
DP00027	6H1W
DP02267	1HRH
DP01382	1ANG
DP00118	1WVW
DP01069	1OYS
DP01060	1LSG
DP00164	1M4C
DP01035	1VF7
DP00555	6Q64
DP00221	6QBP
DP01860	5EGK
DP01801	3MC2
DP01388	4UQF
DP00795	4V2F
DP01101	2PPI

DP02320	2NS7
DP01384	2AAK
DP00973	2FVZ
DP00540	2EO0
DP00163	5X0O
DP00530	6IGT
DP00951	5ZHY
DP01378	1CZD
DP00940	4.00E+61
DP01969	3QIR
DP01787	2ALR
DP02321	4IQA
DP01833	4.00E+16
DP01025	5TDQ
DP00017	6BDC
DP00372	1G62
DP01789	1GSC
DP02078	1GWZ
DP01971	1HMC
DP00521	1IDF
DP00563	1AJA
DP00205	1BVU
DP01559	1CHG
	1RCU
	1RHD
	1RLR
	1K3F
	1N3X
	1NF1
	6ISB
	171L

4I82
3P6A
6HFG
2AW5
3DPA
3DKB
2XSS
3LZ2
3KFW
3APM
3GPN
3HDU
5MQO
3V9G
3SQ3
3IA0
4V2D
4V2E
5C21
5AY9
2OWY
1TIM
2NRO
2HSG
2J5U
2FXO
2EQL
5YBX
6AHO
1XI8
3B8J

Supplementary Table 2. Performance of the pH-independent hydrophobicity model derived by SVM in Supplementary Figure S3 in predicting order-disorder transitions in a C-H plot analysis. Unfolded sequences correctly predicted to be unfolded were classified as true positives.

Measure	pH-independent hydrophobicity SVM analysis
Sensitivity	0.74
Specificity	0.88
Precision	0.90
False Discovery rate	0.08
Accuracy	0.8
F1 Score	0.81
Matthews Correlation Coefficient	0.60
